# Identification of circular RNAs hsa_circ_0140271 in peripheral blood mononuclear cells as a novel diagnostic biomarker for female rheumatoid arthritis

**DOI:** 10.1186/s13018-021-02794-8

**Published:** 2021-10-30

**Authors:** Yufeng Chen, Xianghe Xu, Xuegang Li, Junlong Zhong, Biao Wu, Jie Shang, Ning Jiang, Bin Wang, Shuping Zhong, Huading Lu

**Affiliations:** 1 Department of Orthopaedics, The Fifth Affiliated Hospital of Sun Yat-Sen University, Zhuhai, 519000 Guangdong China; 2 Guangdong Provincial Key Laboratory of Biomedical Imaging, The Fifth Affiliated Hospital, Sun Yat-Sen University, Zhuhai, 519000 Guangdong China; 3 Department of Rheumatology, The Fifth Affiliated Hospital of Sun Yat-Sen University, Zhuhai, 519000 Guangdong China

**Keywords:** Rheumatoid arthritis, Circular RNAs, Biomarker

## Abstract

**Background:**

Rheumatoid arthritis (RA) is a chronic autoimmune disease, which commonly affects women. Accumulating evidence shows that differentially expressed circular RNAs (circRNAs) play crucial roles in the progress of RA. However, the roles of circRNAs in female RA remains unclear. This study explores potential role and diagnostic value of hsa_circ_0140271 from peripheral blood mononuclear cells (PBMC) in female RA.

**Methods:**

Differential expression of circRNAs was determined by RNA-sequencing in PBMC from 4 healthy controls (HC) and 4 RA patients, and we further measured the level of hsa_circ_0140271 in a validation cohort consisting of 47 RA and 47 HC via RT-qPCR. Besides, correlation studies with clinical variables were also examined. What’s more, we performed bioinformatics analysis to predict the potential role of hsa_circ_0140271.

**Results:**

PBMC expression of hsa_circ_0140271 of female RA was significantly higher than that of female HC, and it was positively correlated with antistreptolysin (ASO). Furthermore, the receiver operating characteristic (ROC) curve indicated that hsa_circ_0140271 could distinguish female RA from female HC and female patients with ankylosing spondylitis (AS) or osteoarthritis (OA). Besides, the combined diagnosis anti-cyclic citrullinated peptide (Anti-CCP) + hsa_circ_0140271 could improve diagnostic accuracy with an area under the curve (AUC) of 0.818 to compared with Anti-CCP. Furthermore, KEGG pathway enrichment analysis indicated hsa_circ_0140271 may act as microRNA sponge and participate in fatty acid metabolism pathways.

**Conclusion:**

Hsa_circ_0140271 was likely to be used as a promising diagnostic biomarker for female RA; it may act as microRNA sponge to regulate fatty acid metabolism pathways in RA.

**Supplementary Information:**

The online version contains supplementary material available at 10.1186/s13018-021-02794-8.

## Background

Rheumatoid arthritis (RA) is a systemic autoimmune disease characterized by chronic inflammation and erosive arthrosynovitis, with a prevalence of 0.5%–1% in the worldwide [1, 2]. Like other autoimmune diseases, RA is more prevalent among women, with some studies suggesting as high as 3:1 female-to-male ratio. The etiology of RA is not fully elucidated. However, hormonal change and genetic factors are related to the high incidence of female RA [3]. Women with early menopause are more susceptible to RA [4]. Besides hormone, genes from the X-chromosome are also closely associated with RA. A higher rate of skewed X-chromosome inactivation has been observed in female RA patients [5]. And interleukin 1 receptor-associated kinase (IRAK1) from the X-chromosome has been reported to be related to female predominant in RA [6]. Thus, researches on X-chromosomal genes might be a good way to elucidate the mechanism of female RA etiology.

Circular RNAs (circRNAs) are a class of endogenous RNA, which are characterized by a covalently closed loop structure without a 5′ end cap structure and 3′ end poly (A) tail [7]. Circular RNAs are widely presented in eukaryotic cells and showed cell or tissue-specific expression patterns [8]. Due to loop structure, circular RNAs are resistant to degradation, and this feature has made it possible to use as diagnostic biomarkers or clarify the potential pathogenesis of diseases [9]. Recently, several studies have been demonstrated that circular RNAs are highly relevant to RA [10]. Those studies have explored various roles of circRNAs in RA. However, there are still few reports about circRNAs derived from the X-chromosome and the potential mechanisms of them.

In this study, we aimed to research on circRNAs originate from X-chromosomal and explore the potential role and diagnostic value in female RA. Furthermore, we further evaluated the potential of the candidate circRNAs as a diagnostic biomarker for female patients with RA.

## Methods

### Patient variables

Peripheral blood was collected from a total of 133 participants who were recruited consecutively for this study: 51 patients with RA, 51 healthy controls, 24 female osteoarthritis (OA) and 7 female ankylosing spondylitis (AS) patients. All patients with RA, OA and AS were diagnosed at the Department of Rheumatology and Immunology at The Fifth Affiliated Hospital of Sun Yat-Sen University in 2019. Fifty-one age- and sex-matched healthy controls without renal failure, heart failure or autoimmune disease, and free from other inflammatory conditions, were recruited from the same hospital. All RA patients fulfilled the 2010 rheumatoid arthritis classification criteria. As the standard of classification, disease activity was assessed according to the disease activity score using 28 joint counts (DAS28), DAS28 ≥ 2.6 were allocated to the active-disease cohort, and those with DAS28 < 2.6 were allocated to the stable-disease cohort. Among these, 5 patients were new-onset rheumatoid arthritis (< 6 months of disease duration). The characteristics of RA patients and healthy controls are shown in Table 1. All study protocols were approved by the ethics committee of The Fifth Affiliated Hospital of Sun Yat-Sen University, the Ethics Board Approval number was K62-1, and all participants in this study were informed and signed written consent.

**Table 1:** Laboratory indicators and clinical characteristics of healthy controls and patients with rheumatoid arthritis

Characteristics	RA	HC
Number	47	47
Age (years)^a^	57.13 ± 12.27	60.70 ± 10.89
Sex (M/F)	16/31	16/31
Disease duration (month)^b^	1008.00 (158.40, 1872.00)	NA
Anti-CCP (U/ml)^a^	40.28 ± 15.20	32.34 ± 10.86
DAS28*	4.06 ± 1.14	NA
CRP (mg/L)^b^	25.14 (5.10, 73.40)	3.1 (1.00, 42.65)
ESR (mm/h)^a^	61.17 ± 34.13	33.12 ± 27.18
ASO (µl/mL)^b^	22.94 (11.60, 34.70)	NA
C3 (g/L)^b^	1.29 (1.14, 1.50)	NA
C4 (g/L)^a^	0.27 ± 0.06	NA
Swollen joints^b^	6.00 (2.00, 10.00)	NA
Tender joints^b^	6.00 (3.00, 10.00)	NA
ILD positive (%)	17.02%	NA

In the groups providing PBMCs, there was no significant difference in age (*p* = 0.139, Student's *t*-test) and sex (*p* = 1.000, Chi-square) between RA patients and HC

RA, rheumatoid arthritis; HC, healthy controls; M/F, male/female; anti-CCP, anti-cyclic citrullinated peptide antibodies; DAS28, disease activity score in 28 joints; CRP, C-reactive protein; ESR, erythrocyte sedimentation rate; ASO, antistreptolysin; C3, complement 3; C4, complement 4; ILD, interstitial lung disease; NA, not available

^a^Expressed as mean ± standard deviation

^b^Expressed as the median (25th to 75th percentile)

### Preparation of peripheral blood samples and isolation of RNA and plasma

Peripheral blood samples (6 ml) were collected from each patient and controls subject into EDTA-2 K-containing tubes. Plasma and peripheral blood mononuclear cells (PBMC) were extracted as soon as possible by using the Histopaque-1077 (Sigma-Aldrich, UK) according to the manufacturer’s protocol. Then the plasma was immediately separated and transferred to a fresh RNase-free tube and stored at − 80 °C for Elisa assay. Total RNA was isolated from freshly obtained PBMC using the Total RNA Kit I (Omega Bio-Tek, USA). The concentration and quality of the RNA were assessed by absorbance spectrometry measuring absorbance ratios of A260/A280 and a 260/A230 using a NanoDrop ND-1000 spectrophotometer (ThermoFisher Scientific, USA). Total RNA was kept at − 80 °C or immediately used for reverse transcription.

### RNA-seq analysis

Before RNA-sequencing, the quality of RNA was tested by Agilent 2100 Bioanalyzer (Agilent Technology). 2 μg of RNA sample was taken for RNA-sequencing. RNase R digested and rRNA depleted RNAs were taken to generate the sequencing libraries by using Total RNA-seq (H/M/R) Library Prep Kit for Illumina (Vazyme Biotech) following the manufacturer’s recommendations. The library preparations were sequenced on Hiseq X Ten (Illumina).

### Differential expression analysis

The reads were first mapped to the latest UCSC transcript set using Bowtie2 version 2.1.0 [11], and the gene expression level was estimated using RSEM v1.2.15 [12]. For circRNAs expression analysis, the reads were to the mapped genome using the STAR [13] and DCC [14] was used to identify the circRNAs and to estimate the circRNAs expression. TMM (trimmed mean of M-values) was used to normalize the gene expression. Differentially expressed genes were identified using the edgeR program [15]. Genes showing altered expression with *P* < 0.05 and fold changes ≥ 1.5 were considered differentially expressed.

### Real time-quantitative PCR (RT-qPCR) analysis

A total of 500 ng of each RNA sample was used for reverse transcription using a RevertAid First Strand cDNA Synthesis Kit (Thermo Fisher Scientific, Lithuania). The relative expression of circRNA (Forward Primer: 5′-atcggcttgtaaccacagat-3′, Reverse Primer: 5′-ctcataaagttgttctctcctg-3′) and MED14 (Forward Primer: 5′-cgccaactcttcgttcgatta-3′, Reverse Primer: 5′-gtccacaaacaggatggcttg-3′) was determined on an CFX96TM Touch Real-Time PCR Detection System (BIO-RAD, USA) using EverGreen® Forget-Me-Not qPCR Master Mix (Biotium, USA), as the following PCR thermocycler protocol: Initial Enzyme activation step at 95 °C for 2 min, followed by 40 cycles of 95 °C for 5 s (denaturation), 60 °C for 10 s (annealing) and 72 °C for 10 s (extension). GAPDH (Forward Primer: 5′-agccacatcgctcagca-3′, Reverse Primer: 5′-gcccaatacgaccaaatcc-3′) was used as an internal control.

### ELISA

Plasma concentrations of IL-1α, IL-1β, IL-6, IL-8, TNF-α and IFN-γ were measured by commercially available enzyme linked immunosorbent assay (ELISA) kits (Saicheng Bio-Tek, China) according to the manufacturer’s instruction.

### MicroRNA prediction and KEGG pathway analysis

The microRNAs, potentially related to candidate circRNA, were predicted by the circBank database (http://www.circbank.cn/) and circular RNA Interactome database (https://circinteractome.nia.nih.gov/). Based on predicted microRNAs, KEGG pathway enrichment analyses were performed by DIANA-mirPath (http://www.microrna.gr/miRPathv2). The KEGG pathway analysis was also performed to represent the knowledge on the molecular interaction and reaction networks of the target gene. The KEGG pathway with *P* < 0.05 was considered the significance of the pathway correlations.

### Statistical analyses

Data were statistically described in terms of mean ± standard deviation, medians (quartiles) or proportions when appropriate. All experimental data were analyzed using SPSS software 22.0 (IBM, USA) and GraphPad Prism 8.0 (GraphPad Software, CA). Student’s t test and Mann–Whitney’s U test were employed to compare normally distributed parameters and those with skewed distribution, the Pearson method or the nonparametric Spearman method was used for correlation analysis, and logistic regression analysis was used, as appropriate. Receiver operating characteristic (ROC) curves were performed to evaluate the diagnostic value of circRNA. The area under curve (AUC) was calculated with SPSS software 22.0. *P* < 0.05 was considered to be statistically significant.

## Results

### Screening of female rheumatoid arthritis-associated circRNAs

**Fig. 1:**
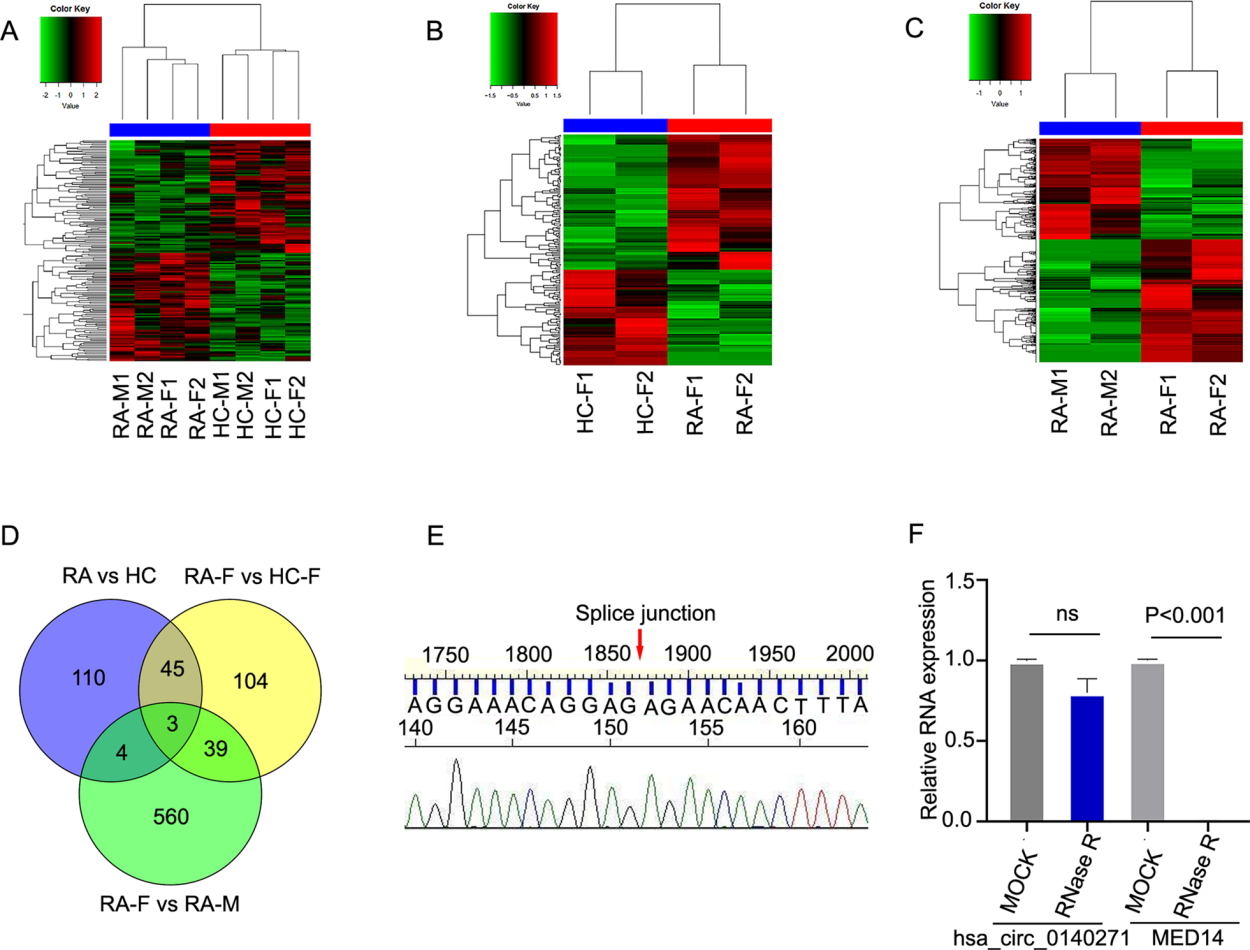
RNA sequencing analysis determined the circRNAs expression profiles and Identification the characterization of circRNAs. (**a**–**c**) Heat map of differentially expressed circRNAs in four RA patients and four healthy controls with age and gender matched (**a**), two RA female patients and two healthy female controls (**b**), two RA female patients and two RA male patients (**c**); ‘Red’ indicates high relative expression, and ‘Green’ indicates low relative expression. (**d**) Venn diagram analysis of three RNA-seq cohort (RA vs HC; RA female vs HC female; RA female vs RA male). (**e**) Sanger sequencing for hsa_circ_0140271. (**f**) Relative expression of hsa_circ_0140271 and mRNA of MED14 in PBMC from RA patients treated with or without RNase R

To explore female RA-associated circRNAs, we firstly collected the total RNA of PBMC samples from 4 RA patients and 4 healthy control subjects with gender and age-matched. Then, we performed RNA sequencing analysis and compared circRNAs expression between rheumatoid arthritis patients and healthy controls. We identified 162 significantly differentially expressed circRNAs with fold changes ≥ 1.5 and a *P* value < 0.05 (Fig. 1a). Next, we used cluster screening to analyze the effect of gender against the background of female healthy samples and male rheumatoid arthritis samples (Fig. 1b, c). This enabled us to further identify female RA-associated circRNAs. As shown in the Venn diagram, we identified three circRNAs (hsa_circ_0140271, hsa_circ_0105101 and hsa_circ_0010474) (Fig. 1d). Among those circRNAs, we were interested in hsa_circ_0140271, which were generated from the MED14 gene located on the X-chromosome and highly expressed in three paired comparisons. To further explore the role of hsa_circ_0140271 in female RA, we designed specific primer to target hsa_circ_0140271 and then performed Sanger sequencing analysis and RT-qPCR. As the results have shown, we verified the back-splice junction of hsa_circ_0140271 through Sanger sequencing analysis (Fig. 1e) and also found hsa_circ_0140271 was resistant to RNase R. While the liner RNA of MED14 was significantly decreased (Fig. 1f). These results confirmed that hsa_circ_0140271 is a stable circRNA molecule and the primer can correctly amplify hsa_circ_0140271.

### Hsa_circ_0140271 was upregulated in PBMC from patients with female rheumatoid arthritis

**Fig. 2:**
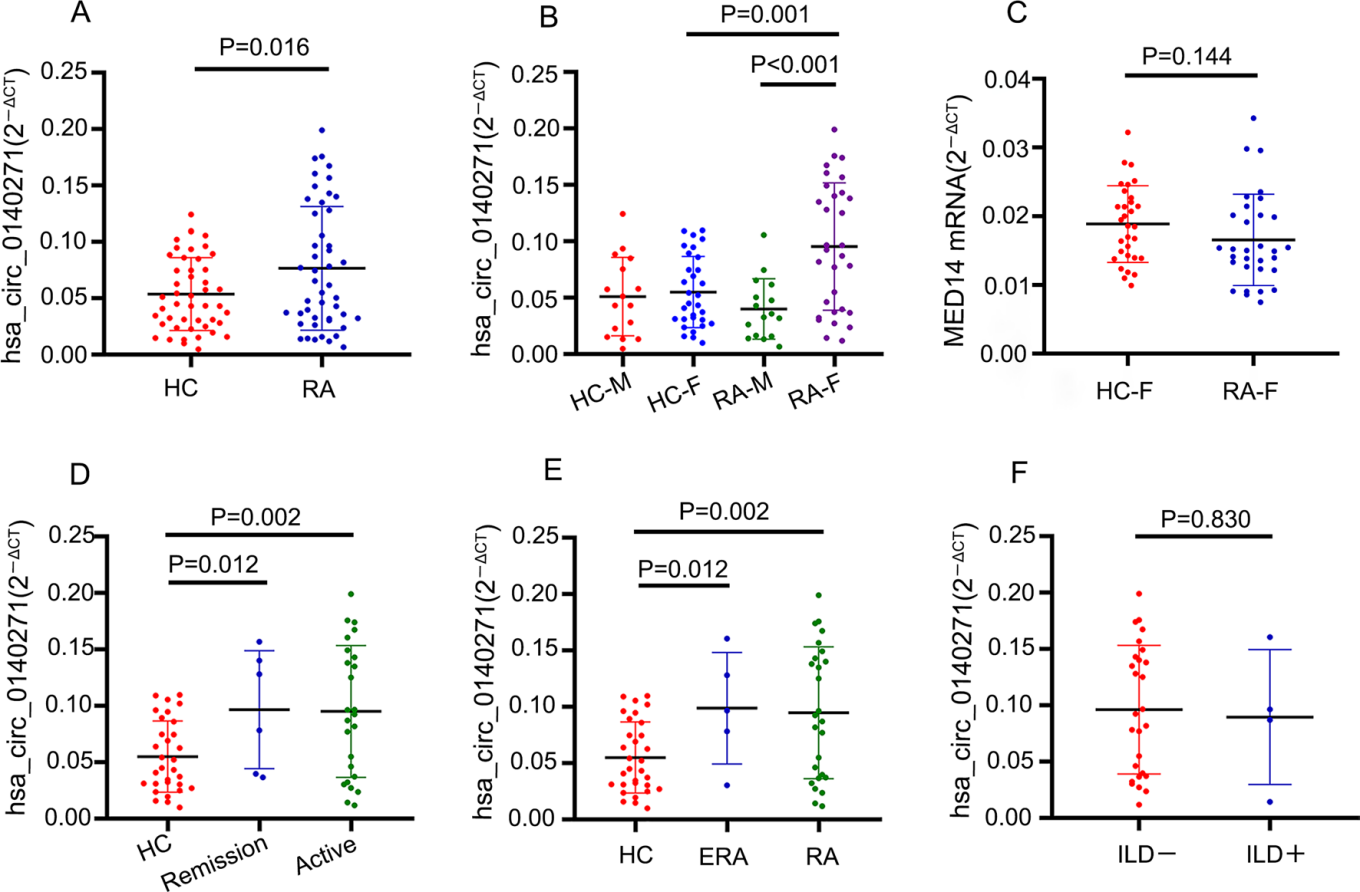
RT-qPCR determined the relative expression level of hsa_circ_0140271 and mRNA of MED14 in PBMC from RA patients and healthy controls. (**a**) Detected expression level of hsa_circ_0140271 in PBMC of RA patients and healthy controls (HC). (**b**) RA patients and HC donors were divided according to gender in each group and then detected expression level of hsa_circ_0140271. (**c**) Detected MED14 mRNA in PBMC of female RA (RA-F) patients and female HC (HC-F) donors. (**d)** Female RA patients were divided according to disease activity of RA and detected expression level of hsa_circ_0140271 in remission and active with comparing to HC. (**e**) Female RA patients were divided according to stage of RA and detected expression level of hsa_circ_0140271 in early RA (ERA) and RA with comparing to HC. (**f**) Detected expression level of hsa_circ_0140271 in ILD positive group (ILD+) and ILD negative group (ILD−)

In order to further analyze hsa_circ_0140271 expression in PBMC from rheumatoid arthritis patients, we continued to collect PBMC samples from 47 RA patients and 47 healthy controls. These two cohorts were also gender and age-matched. The total RNA was also extracted and quantified hsa_circ_0140271 expression by RT-qPCR. As expected, hsa_circ_0140271 was significantly highly expressed in RA samples (Fig. 2a). To assess whether hsa_circ_0140271 would be specifically highly expressed in female RA samples, we stratified RA and healthy control samples according to gender. Consistent with previous results, hsa_circ_0140271 was also significantly highly expressed in female RA samples compared to that in female healthy control samples or male RA samples (Fig. 2b). However, it was not observed any difference in expression of mRNA of MED14 between RA and healthy controls (Fig. 2c).

Next, we analyzed whether hsa_circ_0140271 expression was correlated with the clinical status of female RA patients. According to DAS28 scores, we divided female RA patients into the Remission group (DAS28 < 2.6) and the Active group (DAS28 ≥ 2.6). RT-qPCR results showed the expression of hsa_circ_0140271 was significantly higher in the Remission group and Active group compared to that in the female healthy group, while there was no difference between the Remission group and Active group (Fig. 2d). We also analyzed the effect of disease duration on hsa_circ_0140271 expression and found that the expression of hsa_circ_0140271 was significantly higher in the female early RA (ERA) group (disease duration < 6 months) and RA group (disease duration > 6 months) compared to the female healthy group, but there was also no difference between ERA and RA (Fig. 2e). Finally, we examined hsa_circ_0140271 expression in RA-associated interstitial lung disease (ILD). Among female RA patients, there were four patients who were suffered from ILD, and the expression of hsa_circ_0140271 from those patients was not different from non-ILD patients (Fig. 2f). These data confirmed that hsa_circ_0140271 was specifically highly expressed in PBMC from female RA patients and it was significantly upregulated in the early stage and remission stage of RA, but it was not related to the complication of ILD.

### Hsa_circ_0140271 might serve as a novel diagnostic biomarker for female RA

**Fig. 3:**
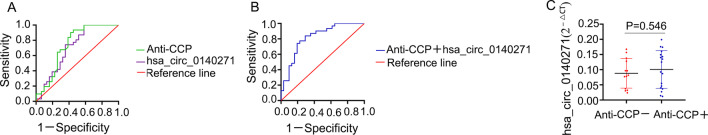
Validation of hsa_circ_0140271 as a potential biomarker for female RA patients. (**a**) The largest AUC was identified for Anti-CCP, followed by hsa_circ_0140271. (**b**) The AUC of combination (Anti-CCP + hsa_circ_0140271) was 0.818, combined diagnosis increased diagnostic accuracy of Anti-CCP. (**c**) The expression level of hsa_circ_0140271 in Anti-CCP positive group (Anti-CCP+) and Anti-CCP negative group (Anti-CCP−)

Based on the results showing that hsa_circ_0140271 was specifically highly expressed in PBMC of female patients with RA, we performed ROC curve analysis to explore the potential utility of hsa_circ_0140271 as a diagnostic biomarker for female RA. According to ROC analysis, the area under curve (AUC) for hsa_circ_0140271 was up to 0.704 (sensitivity = 0.419, specificity = 1), respectively (Fig. 3a; Table 2). While AUC for anti-cyclic citrullinated peptide (Anti-CCP) was 0.738 (sensitivity = 0.581, specificity = 0.903), respectively (Fig. 3a; Table 2). Besides, we also used ROC curve to further evaluate the potential diagnostic value of Anti-CCP combined with hsa_circ_0140271 (Anti-CCP + hsa_circ_0140271). The data suggested that the AUC of the combination was 0.818 (sensitivity = 0.806, specificity = 0.742) (Fig. 3b; Table 2). Finally, stratified by the cutoff value of Anti-CCP, we found that the expression level of hsa_circ_0140271 was no significant difference between the Anti-CCP positive group (Anti-CCP+) and the Anti-CCP negative group (Anti-CCP) (Fig. 3c), which indicated that hsa_circ_0140271 had the same diagnostic efficiency for Anti-CCP positive and Anti-CCP negative patients, so it was suitable for the diagnosis of Anti-CCP negative female RA patients. In a word, these results implied that hsa_circ_0140271 might serve as a novel diagnostic biomarker for female RA and combined diagnosis (Anti-CCP + hsa_circ_0140271) could improve the diagnosis efficacy of female RA.

**Table 2:** ROC curve validates the diagnostic value of differentially expressed hsa_circ_0140271 from female patients with RA

Variables	AUC	SEM	*P* value	95%CI	Sensitivity	Specificity	Cutoff value
hsa_circ_0140271	0.704	0.067	0.006	0.572–0.837	0.419	1.000	0.110
Anti-CCP	0.738	0.738	0.001	0.609–0.867	0.581	0.903	40.719
Anti-CCP + hsa_circ_0140271	0.818	0.054	< 0.001	0.699–0.904	0.806	0.742	NA

### Correlation between hsa_circ_0140271 expression and clinical characteristics

**Fig. 4:**
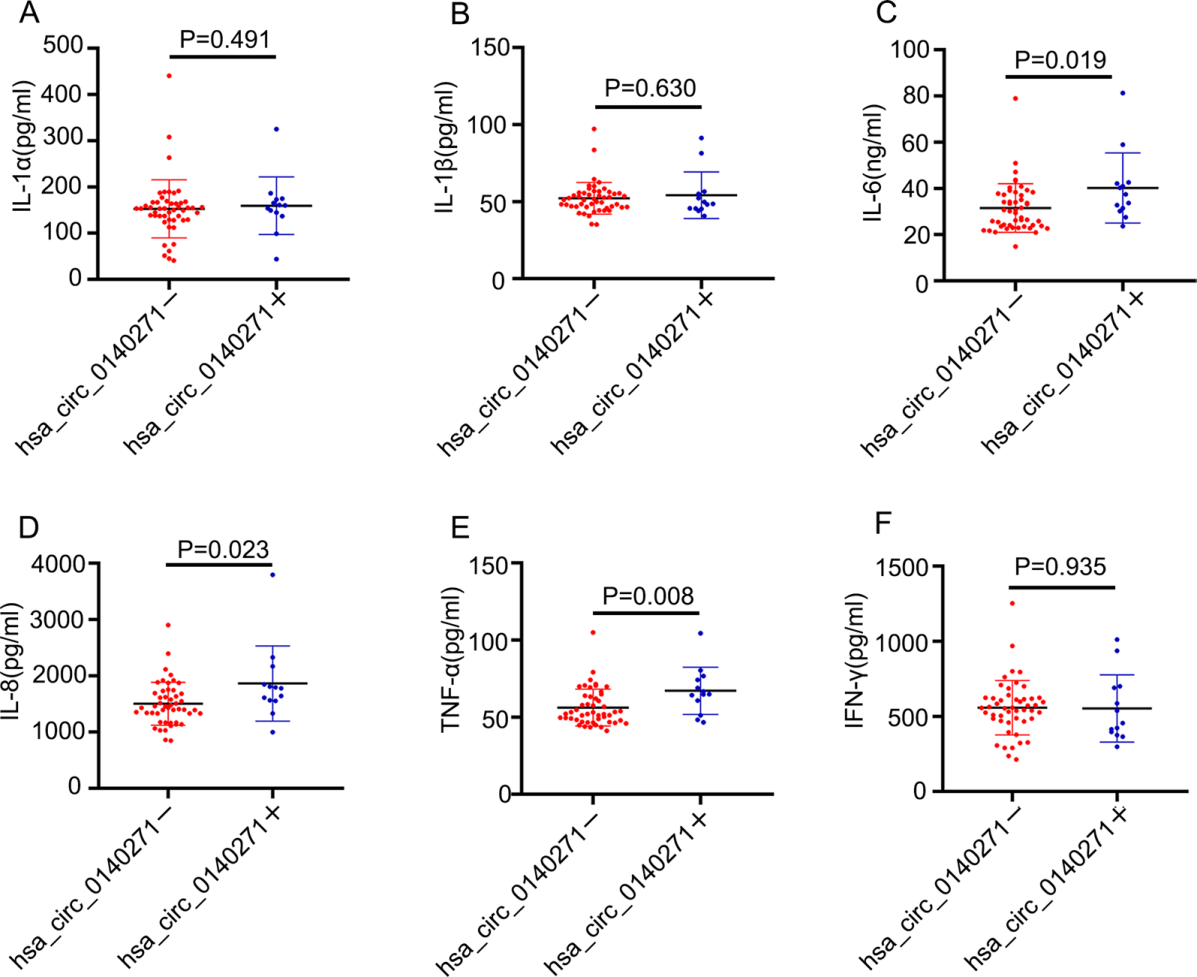
Determined levels of inflammatory factors in plasma of female RA patients and female HC donors. 31 female RA patients and 31 female HC donors were divided according to cutoff levels of hsa_circ_0140271. Detected levels of IL-1α (**a**), IL-1β (**b**), IL-6 (**c**), IL-8 (**d**), TNF-α (**e**) and IFN-γ (**f**) in hsa_circ_0140271 positive group (hsa_circ_0140271+) and hsa_circ_0140271 negative group (hsa_circ_0140271−)

Since some cytokines, such as IL-1α, IL-1β, IL-6, IL-8, TNF-α and INF-γ, were contributed to the pathology of RA [16], we analyzed the relationship between hsa_circ_0140271 expression level in PBMC and those cytokines or clinical features of 31 female RA patients (Table 3). Data showed that the levels of hsa_circ_0140271 were correlated with ASO (*r* = 0.416, *P* = 0.020). Based on cutoff values of hsa_circ_0140271, we divided female RA patients into the hsa_circ_0140271 positive group (> 0.110) and negative group (< 0.110). Then we detected IL-1α, IL-1β, IL-6, IL-8, TNF-α and INF-γ expression in plasma from both groups. Unfortunately, there was no difference in the level of those inflammatory factors between the two groups (Additional file 2: Figure S1). However, when dividing female RA and female healthy controls according to the cutoff values of hsa_circ_0140271, the results showed that IL-6, IL-8 and TNF-α expression was higher in the hsa_circ_0140271 positive group (Fig. 4c–e), while there was no significant difference in the level of IL-1α, IL-1β and INF-γ between two groups (Fig. 4a, b, f).

**Table 3:** Correlation between hsa_circ_0140271 expression and clinical characteristics

Characteristics		*N*	*R*	*P*
IL-1α (pg/ml)^b^	153.59 (112.47, 166.71)	31	0.085	0.649
IL-1β (pg/ml)^b^	48.20 (45.56, 55.00)	31	0.018	0.924
TNF-α (pg/ml)^a^	64.75 ± 15.18	31	0.045	0.811
IFN-γ (pg/ml)^a^	520.60 ± 248.14	31	0.017	0.928
IL-6 (ng/ml)^b^	37.29 (31.43, 40.99)	31	− 0.058	0.757
IL-8 (pg/ml)^b^	1641.11 (1342.50, 1875.83)	31	0.049	0.793
CRP (mg/L)^b^	18.10 (5.10, 46.30)	31	− 0.231	0.210
ESR (mm/h)^a^	60.58 ± 34.74	31	0.005	0.980
ASO (lu/mL)^b^	25.04 (10.58, 43.73)	31	0.416	0.020*
C3 (g/L)^a^	1.25 ± 0.23	30	0.097	0.611
C4 (g/L)^a^	0.27 ± 0.07	30	− 0.349	0.059
DAS28-CRP^a^	4.05 ± 1.23	31	0.043	0.817
Disease duration (month)^b^	108.00 (24.00, 192.00)	31	− 0.350	0.054
Swollen joints^b^	8.00 (2.00, 10.00)	31	− 0.120	0.521
Tender joints^a^	7.03 ± 4.79	31	− 0.051	0.787

### Hsa_circ_0140271 might regulate RA progression by modulating fatty acid metabolism pathways

**Fig. 5:**
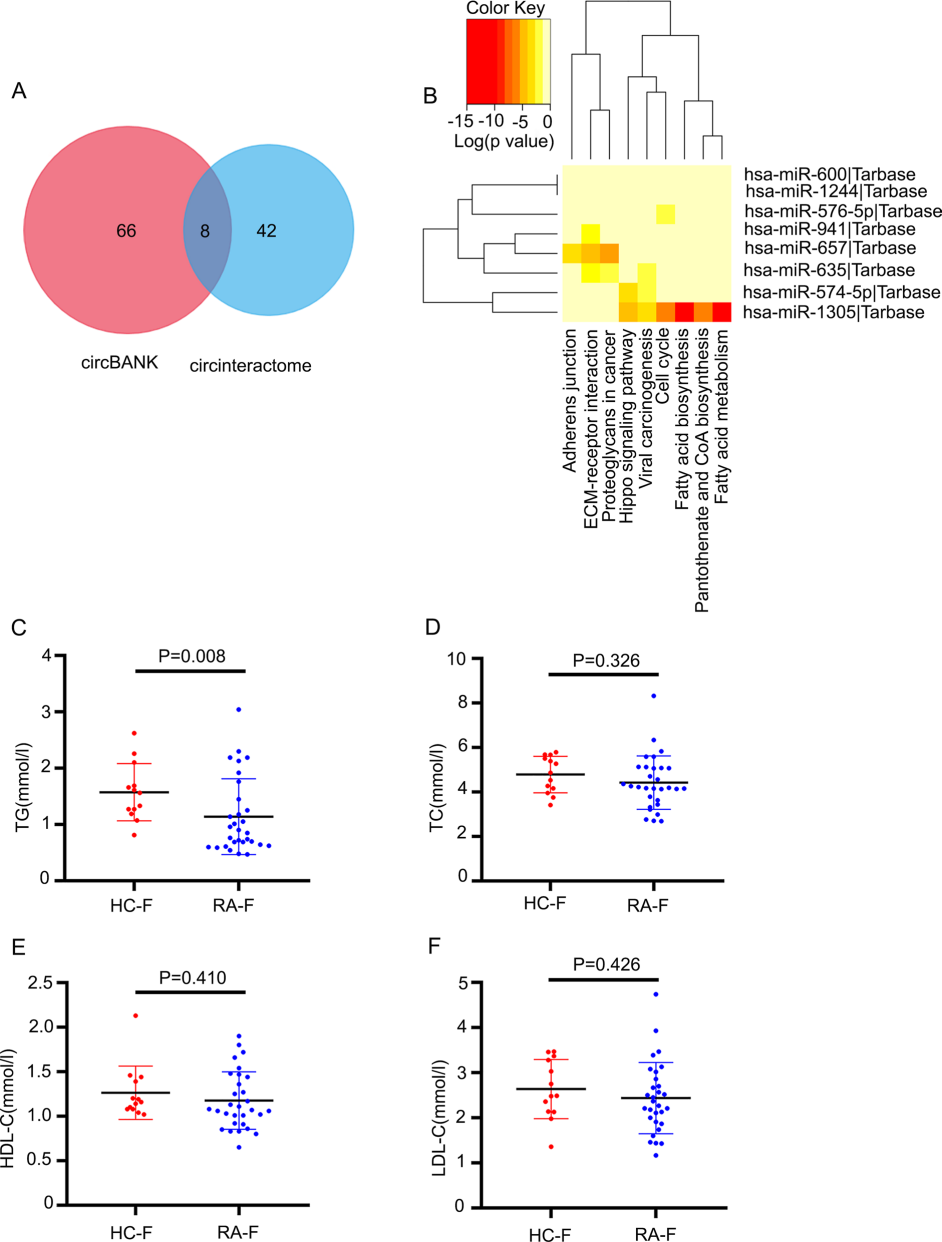
Prediction role of hsa_circ_0140271 in female RA patients. (**a**) Venn Diagram showed the number of potentially hsa_circ_0140271 associated microRNAs. (**b**) KEGG pathway analysis provided potential signaling pathways regulated by hsa_circ_0140271. (**c**–**f**) The levels of TG, TC, HDL-C and LDL-C of plasma were detected in female RA patients and female HC donors

Accumulating pieces of evidence have been shown that circRNAs play critical role in the pathogenesis of diseases through function of microRNA sponge [17, 18]. To elucidate the role of hsa_circ_0140271 in RA, we predicted hsa_circ_0140271 related microRNA using the circBank database (http://www.circbank.cn/) and circular RNA Interactome database (https://circinteractome.nia.nih.gov/) (Fig. 5a). By analyzing these two databases, it was shown eight microRNAs might closely relate to hsa_circ_0140271, which were has-miR-600, has-miR-1244, has-miR-576-5p, hsa-miR-941, has-miR-657, has-miR-635, has-miR-574-5p and has-miR-1305. Then, to further predict the function of hsa_circ_0140271, we performed KEGG analysis based on predicted microRNAs using DIANA-mirPath (http://www.microrna.gr/miRPathv2) (Fig. 5b). According to the analysis, we found 9 enriched KEGG terms. Intriguingly, we found that no terms were related to inflammation. However, we found 3 terms that were related to lipid metabolism, which were “Fatty acid biosynthesis”, “Pantothenate and CoA biosynthesis” and “Fatty acid metabolism”. This analysis indicated function of hsa_circ_0140271 might be associated with lipid metabolism. Recently, growing evidences suggest that lipid metabolism has been found to contribute to RA progress, especially when correlated with chronic inflammation [19]. A previous study showed the serum levels of triglycerides (TG) and total cholesterol (TC) were higher in pre-RA peoples than in controls [20]. However, contrary to this results, the serum levels of TG and TC were lower in the RA patients than in controls [21]. And low level of TC and TG seems to be related to inflammation of RA [22]. In women, a high serum level of TG, but not TC, increased the risk of RA. Although lipid metabolism is complex and even showed a paradox effects on RA, it plays a potential risk factor or a mediator in female RA progress. In order to verify our hypothesis, we checked lipid indicators of female RA patients (Fig. 5c–f). It was found that the expression of TG from female RA patients was lower than female controls group (Fig. 5c), while it was not observed any difference in the expression of TC, HDL-C and LDL-C between female RA and female healthy controls. Taken together, these results suggested that hsa_circ_0140271 might regulate RA progression by modulating fatty acid metabolism pathways through the function of microRNA sponge.

### Hsa_circ_0140271 was a potential biomarker in discriminating female RA from female OA or AS

**Fig. 6:**
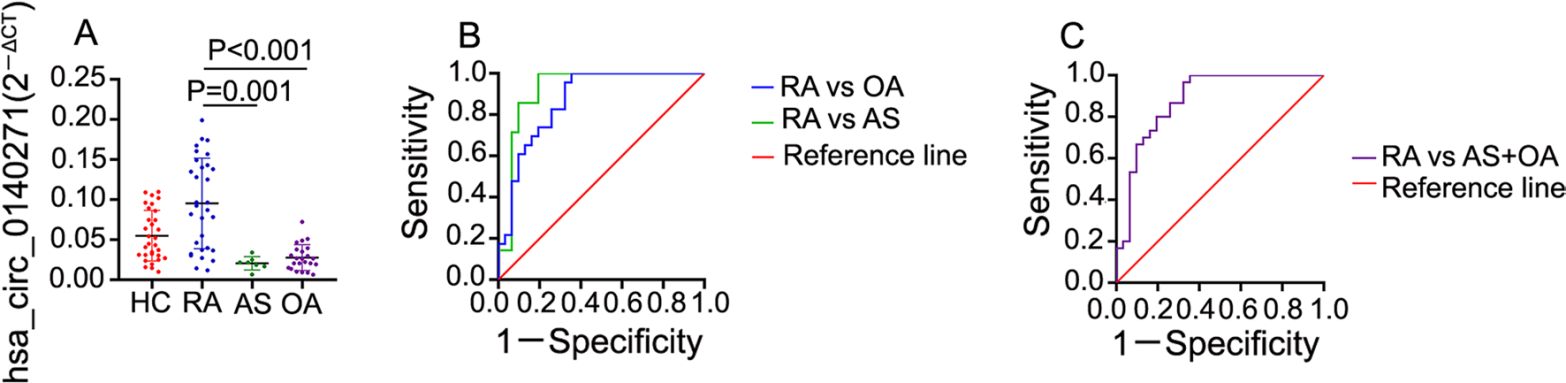
Validation of the specific diagnostic values of hsa_circ_0140271 in female RA patients. (**a**) RT-qPCR confirmed hsa_circ_0140271 expression in female RA patients, female OA patients, female AS patients and female HC donors. (**b**, **c**) ROC curve analysis of hsa_circ_0140271 in female patients with RA compared with OA or AS

Since the autoimmune disease is predominant in female patients [3], we also analyzed hsa_circ_0140271 expression in PBMC from female patients with osteoarthritis (OA) and ankylosing spondylitis (AS), the characteristics of disease controls are shown in Additional file 1: Table S1. We recruited 24 female OA patients and 7 female AS patients and RNA of those patients' PBMC was also extracted to perform RT-qPCR. The RT-qPCR analysis demonstrated that hsa_circ_0140271 expression from female OA and AS was significantly lower than in female RA, even lower than that from female healthy controls (Fig. 6a). Then, we performed ROC curve analysis to evaluate the potential diagnostic efficiency of hsa_circ_0140271 in discriminating RA from OA or AS. ROC curve from patients with RA and AS showed AUC was 0.922 (sensitivity = 1, specificity = 0.806). When distinguishing RA patients from OA patients, AUC was 0.868 (sensitivity = 1, specificity = 0.645) (Fig. 6b; Table 4). While discriminating RA from disease controls (AS + OA). AUC was  0.818 (sensitivity = 1, specificity = 0.645) (Fig. 6c; Table 4). Those analyses implied that hsa_circ_0140271 was specifically highly expressed in female RA patients and might be a potential biomarker in discriminating female RA from famale OA or AS.

**Table 4:** ROC curve analysis of the confirmed hsa_circ_0140271 as RA female specific diagnosis

Variables	AUC	SEM	*P* value	95%CI	Sensitivity	Specificity	Cutoff value
hsa_circ_0140271 (RA vs. AS)	0.922	0.045	< 0.001	0.787–0.984	1	0.806	0.034
hsa_circ_0140271 (RA vs. OA)	0.868	0.048	< 0.001	0.748–0.945	1	0.645	0.072
hsa_circ_0140271 (RA vs. AS + OA)	0.818	0.045	< 0.001	0.772–0.950	1	0.645	0.072

## Discussion

Rheumatoid arthritis is a chronic autoimmune disease, which can cause cartilage and bone damage. Early diagnosis is key to optimal therapeutic success [23]. Currently, increasing studies have been demonstrated that non-coding RNAs are involved in several disease progress and may act as biomarkers for diagnosis [10, 24, 25]. In this study, we found that hsa_circ_0140271 had the potential to serve as a novel diagnostic biomarker for female RA and combined diagnosis (Anti-CCP + hsa_circ_0140271) could improve the diagnosis efficiency of female RA.

Like other autoimmune diseases, women are more frequently to be affected by RA, with a female-to-male ratio of about 3:1 [3]. Genes from the X-chromosome are factors that contribute to the predominance of RA in women [26]. Normally, a female karyotype is comprised of two X-chromosomes, which are originated from each parent. During the early stage of embryogenesis, one of X-chromosomes is randomly silenced. However, 15% of the genes would be escaped from silencing and X-chromosomes inactivation (XCI) is associated with female RA [5, 26, 27]. One mechanism of XCI is DNA methylation, which is potentially related to circular RNA biogenesis and RA [27–29]. And the generation of circRNAs was also regulated by DNA methylation [28]. In this study, we performed RNA sequencing analysis and found hsa_circ_0140271 expression might be related to female RA. We also detected the X-chromosomal gene-MED14 expression, from which hsa_circ_0140271 was generated. However, there was no difference in MED14 mRNA expression between the female RA group and the female healthy controls. These results indicated that the difference of hsa_circ_0140271 expression was not caused by the gene of MED14 itself, while it may be closely associated with female RA or other things, like DNA methylation. And it is needed to further explore the mechanism of generation of hsa_circ_0140271 in female RA.

It is well accepted that early diagnosis and assessment of disease activity are favorable outcomes in RA [30–32]. In this study, we observed that there was no difference in hsa_circ_0140271 expression between the remission and activity group in female RA patients. Furthermore, there was also no difference in hsa_circ_0140271 expression between early-stage RA (ERA) and RA group in female RA patients, which meant hsa_circ_0140271 was likely to become a diagnostic biomarker for ERA and remission stage of RA. Compared to female healthy controls, AS or OA group, ROC curve analysis also showed that the sensitivity of hsa_circ_0140271 was all 1, which implied that it might have a good diagnosis value for female RA. We also detected hsa_circ_0140271 expression in one of RA complications-interstitial lung disease (ILD). In this analysis, we also did not find any difference in hsa_circ_0140271 expression between ILD and non-ILD group. These analyses indicated that hsa_circ_0140271 could not serve as a biomarker for assessing complications of ILD. However, it might be a potential biomarker for ERA and remission RA.

RA is a chronic inflammatory disease, and proinflammation factors play important role in RA progress [16]. In this study, we detected IL-1α, IL-1β, IL-6, IL-8, TNF-α and INF-γ levels in plasma among female RA and female HC. We analyzed the relationship between hsa_circ_0140271 expression level and those cytokines or clinical features of 31 female RA patients. Data showed that the levels of hsa_circ_0140271 were positively correlated with ASO. It is suggested that female RA patients with high expression of hsa_circ_0140271 were more likely to be infected with hemolytic streptococcus. Based on cutoff values of hsa_circ_0140271 expression, there was no difference in those factors level between hsa_circ_0140271 positive and negative group among female RA patients, but it was observed IL-6, IL-8 and TNF-α levels were higher in the hsa_circ_0140271 positive group when calculating female RA patients and female healthy subjects together. Regarding these results, we estimated that the limited sample size affected the results in female patients, and with increasing sample size there would be also different in those factors level between the hsa_circ_0140271 positive and negative group among female RA patients. Interestingly, it has been found that levels of IL-6, IL-8 and TNF-α in plasma were associated with the early stage of RA patients [33, 34]. These results implied that hsa_circ_0140271 might be related to the onset or pathology of early-stage of RA. And combining those analyses and previous analyses of hsa_circ_0140271 expression in early-stage RA and RA group, we estimated that hsa_circ_0140271 might become a diagnostic biomarker of the early-stage female RA.

To further explore the function of hsa_circ_0140271, we performed bioinformatic analysis. According to microRNA sponge theory, we predicted some microRNAs related to hsa_circ_0140271 and then performed KEGG pathway analysis to found some signaling pathways potentially regulated by hsa_circ_0140271. Contrary to our expectation, there were no pathways related to inflammation. However, we detected three fatty acid-related pathways, such as fatty acid biosynthesis, pantothenate and CoA biosynthesis and fatty acid metabolism. It has been demonstrated that fatty acids play a role in various RA processes, especially inflammation [35]. Previously, preclinical RA and early patients have demonstrated that normal or slightly increased TG, TC LDL and decreased HDL [36, 37]. As hsa_circ_0140271 might be related to early-stage RA, we detected increased TG in hsa_circ_0140271 positive group of female RA. People who have higher TG would be more easily to develop RA in their life [20]. And high TG is also correlated with systemic inflammation in RA. However, contrary to this study, Carl has reported that TC, but not TG, was a risk factor for female RA. The difference of results between our and Carl’s study is caused by methodological difference, such as included sample size or time of disease progression. Taken together, we hypothesize that hsa_circ_0140271 might regulate fatty acid metabolism in the processes of female RA, and it is needed to explore the role of hsa_circ_0140271 in regulating fatty acid metabolism in female RA in future.

In general, we suggest hsa_circ_0140271 as a potential diagnostic biomarker for female RA. However, there are some limitations that should be considered in this study. Firstly, the sample size of validation cohort was relatively small, and the samples were recruited from only one hospital, which may limit the universality of the results. Secondly, all patients with RA and healthy controls in this study were Chinese; additional studies in other ethnic groups are needed to confirm these findings. Thirdly, the specific role of hsa_circ_0140271 in RA pathogenesis was not fully explored. In this study, we recruited 51 RA patients and 51 healthy controls. As mentioned above, the level of inflammation factors, such as IL-6, IL-8 and TNF-α, was shown to be different when calculating female RA patients and female healthy subjects together. However, no difference was seen in female RA patients alone. Therefore, a large number of cohort study would be needed to be performed in the future study. Besides, we predicted the function of hsa_circ_0140271 based on the theory of microRNA sponge. Except for function of microRNA sponge, circular RNAs also interact with functional protein to regulate transcription activity in cells [38]. Thus, it should carefully explore the function of hsa_circ_0140271 via more relative experiments in the future.

## Conclusion

This study aimed to explore X-linked circRNA to elucidate its potential role in the diagnosis of female RA. Our results have provided evidences that hsa_circ_0140271 was likely to be used as a promising diagnostic biomarker for female RA. It may act as microRNA sponge to play an important role in regulating the processes of fatty acid metabolism.

## Supplementary Information

**Additional file 1.** Clinical description of patients who participated in the study.

**Additional file 2.** Determined levels of inflammatory factors in plasma of female RA patients.

## Data Availability

The datasets used and/or analyzed during the current study are available from the corresponding author on reasonable request.

## Abbreviations

RA: Rheumatoid arthritis
circRNAs: Circular RNAs
PBMC: Peripheral blood mononuclear cells
HC: Healthy controls
ASO: Antistreptolysin
ROC: Receiver operating characteristic
Anti-CCP: Anti-cyclic citrullinated peptide
AS: Ankylosing spondylitis
OA: Osteoarthritis
DAS28: Disease activity score using 28 joint counts
ILD: Interstitial lung disease
