# Supplementary material for: Identification of circular RNAs hsa_circ_0140271 in peripheral blood mononuclear cells as a novel diagnostic biomarker for female rheumatoid arthritis
Source: J Orthop Surg Res. 2021 Oct 30;16:647. doi: 10.1186/s13018-021-02794-8 (PMC8557002; doi:10.1186/s13018-021-02794-8)
Supplement: Supplementary file 1 — Additional file 1. Clinical description of patients who participated in the study. [file 13018_2021_2794_MOESM1_ESM.docx]

| **Supplementary Table 1**. Clinical description of patients who participated in the study. | | | |
| --- | --- | --- | --- |
| Characteristics | RA | AS | OA |
| Number | 31 | 7 | 24 |
| Age(years) | 54.29±12.31 | 29.71±6.21 | 60.00±5.48 |
| Sex(female) | 100% | 100% | 100% |
| Hsa_circ_0140271 levels | 0.095±0.056 | 0.021±0.008 | 0.028±0.016 |
