# Supplementary material for: Identification of circular RNAs hsa_circ_0140271 in peripheral blood mononuclear cells as a novel diagnostic biomarker for female rheumatoid arthritis
Source: J Orthop Surg Res. 2021 Oct 30;16:647. doi: 10.1186/s13018-021-02794-8 (PMC8557002; doi:10.1186/s13018-021-02794-8)

**Supplementary figure legends**

**Supplementary Fig 1. Determine levels of inflammatory factors in plasma of female RA patients.** 31 female RA patients were divided according to the Cutoff value of hsa_circ_0140271. Detected levels of IL-1α (A), IL-1β (B), IL-6 (C), IL-8(D), TNF-α (E) and IFN-γ (F) in hsa_circ_0140271 positive group (hsa_circ_0140271 ＋ ) and hsa_circ_0140271 negative group (hsa_circ_0140271－).


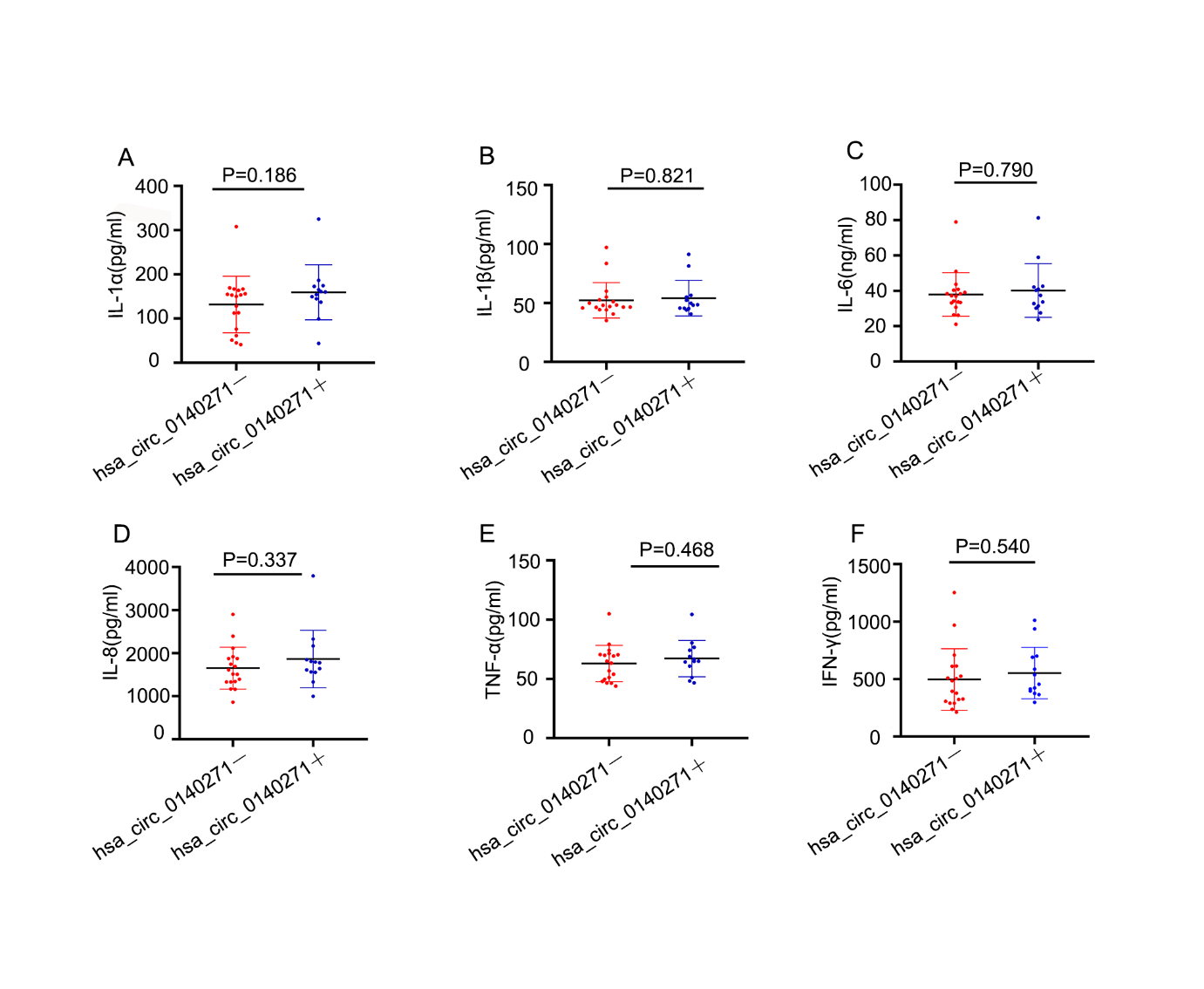

Supplement: Supplementary file 2 — Additional file 2. Determined levels of inflammatory factors in plasma of female RA patients. [file 13018_2021_2794_MOESM2_ESM.docx]
